# Lessons from COVID-19: Towards a global pandemic security partnership

**DOI:** 10.7189/jogh.11.03028

**Published:** 2021-01-30

**Authors:** Christabel Sefa, Nana Sefa

**Affiliations:** 1 Michigan Center for Global Health Equity, Michigan Medicine, University of Michigan, Ann Arbor, Michigan, USA; 2 Division of Emergency Critical Care, Department of Emergency Medicine, University of Michigan, Ann Arbor, Michigan, USA

In the entire 20^th^ century, there were three pandemics [1]. By contrast, only 20 years into the 21^st^ century, the world has already seen 2 deadly global pandemics and a disproportionate number of increasingly fatal epidemics [2-4]. The current COVID-19 pandemic has rapidly impacted almost every country on the globe, infecting 71.6 million and resulting in 1.6 million deaths as of December 12, 2020 [5,6].

This swift succession of epidemics and pandemics over the past 20 years, marked by increasing scale, frequency, and mortality is at odds with global health systems that are more sophisticated today. This trend can be partially explained by increased globalization and frequency of international travel [7]. In addition, several environmental and social factors germane to life in the 21^st^ century, such as population growth and urbanization, have increased the capacity of novel infectious diseases to spread rapidly among humans. Furthermore, epidemics can go undetected for a long time due to either lack of knowledge that a particular trend of illness is the beginning of a potential epidemic or the fear of blame, which leads to delayed reporting of threatening epidemics to the global community. There are also severe economic repercussions of the mitigation strategies necessary to stop the spread of novel diseases. In addition, the sudden disruption of normal economic activity from pandemics leads to uncertainty and destabilization that have significant security implications for most nations.

If we are to overcome pandemic threats with the speed and efficacy that modern medicine evokes, then proactive, collective preparation to prevent and control the spread of contagious infectious diseases should become our new normal. COVID-19 has shown that next pandemic could start *anywhere* but affect everyone within weeks. The random nature of the origins of a pandemic are the very reason why all countries should be involved in creating a system to respond to epidemics of pandemic potential regardless of location. We therefore propose the establishment of a *Global Pandemic Security Partnership* to address the coordination failures that allowed COVID-19 to become a pandemic, and to serve as a taskforce to tackle future pandemic threats.

## MISSION AND SCOPE

The primary mission of the Global Pandemic Security Partnership (GPSP) would be to prevent future pandemics by providing a platform to aggressively respond to pandemic-threatening epidemics around the world with the required systems and resources. Its secondary aim would be to provide a platform to coordinate a response to any future pandemics. The goal is to invest in early mitigation systems that would cost less than the economic impact of a full-scale pandemic.

## OBJECTIVES

The objectives of the GPSP are as follows:

*1. Foster early global notification and response:* Early and effective action is needed at the beginning of any epidemic, especially if it involves a condition that is not endemic to a particular location. For instance, when the COVID-19 outbreak started, the early global decision to aggressively test and quarantine all travelers from hotspots may have slowed the spread significantly but would have been accompanied by significant economic costs. Therefore, the GPSP would support countries to undertake effective early detection and containment of pandemic-threatening epidemics using a pool of technical and financial resources. This support for early containment measures would also shift the culture from naming and shaming to emphasizing timely global notification, as well as swift, collective, and supportive action to contain any threats. The technical resources of the GPSP would be structured around a Global Pandemic Intelligence Service, an elite team of epidemiologists and outbreak investigators who would continuously monitor epidemics around the world and be ready to be deployed at short notice to sites of concern. This team would also build the capacity of GPSP member countries to investigate, identify, track and control disease outbreaks before they become major epidemics.

*2. Facilitate vaccine distribution:* As researchers race to develop vaccines for COVID-19, there is ongoing discussion about how the vaccines would be distributed. Many developing countries were skeptical about allowing vaccine clinical trials to occur in their countries due to concerns that their citizens would be used as subjects for trials but would be unable to get the vaccine in a global scramble to purchase doses. Some countries had placed orders with multiple companies even before a viable vaccine was developed and approved. This raises the question as to whether the vaccine would be distributed around the globe to those most at risk or to the highest bidders. Various foundations have stepped in and allocated some funds for vaccine distribution, but these interim measures are not part of any global policy or approach that can guarantee that the world’s most vulnerable are vaccinated and protected expeditiously. The GPSP would therefore build on past vaccine distribution experiences to develop an equitable and a priori vaccine distribution strategy, unique to each pandemic, that relies on sound epidemiologic metrics and results in the highest possible utility and worldwide adoption. Buy-in for this strategy, where others have failed in the past, will be sought based on scientific data that shows the places on the globe where stopping the pandemic would have the largest global impact on stopping the spread. This evidence-based strategy would bring transparency and accountability to the vaccine distribution process and provide the assurance needed for vaccines to be tested on a sufficiently diverse population.

**Figure:**
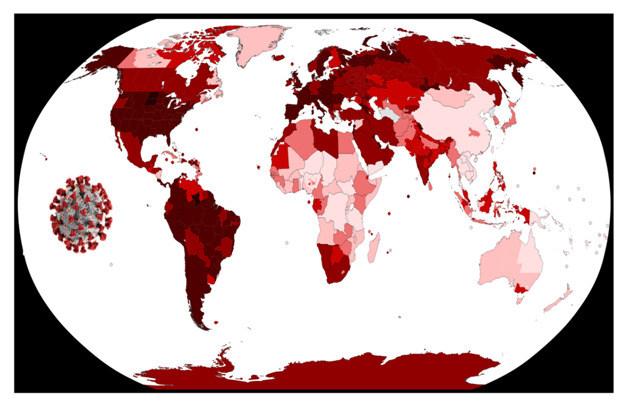
Photo: Global Distribution of COVID-19 as of January 9, 2021 (from https://pxhere.com/en/photo/1608792 and https://commons.wikimedia.org/wiki/File:COVID-19_Outbreak_World_Map_per_Capita.svg).

*3. Provide global guidelines on pandemic response:* The COVID-19 pandemic has revealed the flaws in the current global mechanism of early collation of data on pandemic-threatening epidemics and issuance of guidelines to countries to reduce the spread. This gap highlights the need for mechanisms to quickly develop and issue effective policies and guidelines on an epidemic or pandemic response. This objective will therefore focus on providing universal, unbiased, evidence-based, and independently generated policies and guidelines, immune from the local politics of any particular country, that member countries can adapt and use the contain the spread of epidemics.

## GOVERNANCE AND FUNDING

Existing global organizations fulfill a range of roles, from undertaking overall global health outreach and coordination, to providing ad hoc triaging and diagnosing of outbreaks. The purpose of the GPSP is not to duplicate these functions, but rather to serve as a strategic, coordinating platform that would provide a holistic global response to pandemic threats. We therefore propose that the GPSP be an independent, non-profit organization that would work in close collaboration with existing international health organizations. The governing board of the GPSP should include a broad range of stakeholders, such as national Governments, international organizations, corporations, and other global funds. Sources of financing for GPSP’s activities could include: (i) contributions from member states pegged at a small share of their GDP; (ii) donations from international foundations and global funds; and (iii) donations and taxes from businesses and industries.

## CONCLUSION

Recent strides have been made to improve quality of life for all populations. Global initiatives such as the Millennium Development Goals have made a noticeable dent in maternal and infant mortality, and pockets of global health collaborations continue to chip away at disparities in access to affordable health care. Pandemics threaten these hard-won gains, making pandemic prevention imperative. A major obstacle to the GPSP is getting political will when the world is confronted with recessions and increasing nationalism. As such, the economic rationale to forming this partnership must be strong enough to attract buy-in from a broad coalition of world leaders. Preventing the next pandemic is akin to preventing the next recession, a potential cause of global instability, which provides a strong economic and national security justification for a global investment to prevent the next pandemic. We call on all Governments, international organizations, global foundations, and corporations to join a multi-stakeholder process to *Advocate, Conceptualize, Design, and Establish* the Global Pandemic Security Partnership.

## References

[1] Morens DM Taubenberger JK The mother of all pandemics is 100 years old (and going strong)! Am J Public Health. 2018;108:1449-54. 10.2105/AJPH.2018.304631 30252528 PMC6187799

[2] SARS | Basics Factsheet | CDC. Available: https://www.cdc.gov/sars/about/fs-sars.html. Accessed: 17 September 2020.

[3] 2009 H1N1 Pandemic (H1N1pdm09 virus) | Pandemic Influenza (Flu) | CDC. Available: https://www.cdc.gov/flu/pandemic-resources/2009-h1n1-pandemic.html. Accessed: 17 September 2020.

[4] 2014-2016 Ebola Outbreak in West Africa | History | Ebola (Ebola Virus Disease) | CDC. Available: https://www.cdc.gov/vhf/ebola/history/2014-2016-outbreak/index.html. Accessed: 17 September 2020.

[5] Dong E Du H Gardner L An interactive web-based dashboard to track COVID-19 in real time. Vol. 0. Lancet Infect Dis. 2020;20:533-4. 10.1016/S1473-3099(20)30120-1 32087114 PMC7159018

[6] COVID-19 Map - Johns Hopkins Coronavirus Resource CenterAvailable: https://coronavirus.jhu.edu/map.html. Accessed: 8 November 2020.

[7] Goodman WC Transportation by air: Job growth moderates from stellar rates. Mon Labor Rev. 2000;123:34-47.

